# Association of Dental Caries in Primary Teeth With HLA Class II Gene Among Syrian Children

**DOI:** 10.7759/cureus.53081

**Published:** 2024-01-27

**Authors:** Nassouh Malas, Chaza Kochaji, Chadi Soukkarieh, Mohammed Bashier Almonakel, Hasan Alzoubi

**Affiliations:** 1 Pediatric Dentistry, Damascus University, Damascus, SYR; 2 Animal Science, Damascus University, Damascus, SYR

**Keywords:** dna sequencing, primary teeth, dental caries, hla class ii alleles, s-ecc

## Abstract

Background and objectives

Numerous studies have shown that there is evidence that genetic factors contribute in different ways to the occurrence of caries lesions, and the multiple differences in the human leucocytes antigen (HLA) gene patterns play an important role in the body's immune response. Therefore, this study aimed to evaluate the association between some HLA class II alleles (HLA-DR4, HLA-DQ2, HLA-DQ4) and early childhood caries (ECC) occurrence in Syrian children.

Materials and methods

The study included 80 children aged three to six, divided into two groups: Group 1 (n = 40) being severe early childhood caries (S-ECC) children (deft ≥ 10) and Group 2 (n = 40) being free caries children (deft = 0). The genomic DNAs were extracted and collected by taking a buccal swab using a sterile metal strip and were amplified by polymerase chain reaction-single specific primer (PCR-SSP) and then HLA-typing was performed for all alleles.

Results

There were no statistically significant differences in the frequency of occurrence of S-ECC, HLA-DR4, and HLA-DQ2 alleles (p = 0.626, 0.256 respectively), while the incidence of S-ECC was associated with the DQ4 allele (p = 0.012).

Conclusion

HLA class II molecules may play a crucial role in predisposing to ECC, since positive HLA-DQ4 may enhance the chance of developing ECC. However, no association were found between (HLA-DR4 and HLA-DQ2) and ECC.

## Introduction

According to the World Health Organization, dental caries affects 60-90% of children and most adults worldwide, making it a serious health issue. Dental caries is a multifactorial, chronic, and complicated disease that is highly widespread in both developed and developing countries [1]. Dental caries affects 2.43 billion people worldwide who have permanent teeth and 620 million children who have deciduous teeth, making it one of the most prevalent and common disorders affecting humans [2]. Early childhood caries (ECC) incidence is crucial because it triggers the development of caries in permanent teeth [3].

Despite the great developments that took place in the last century in terms of understanding dental caries, and dealing with it in diagnosis, treatment, and prevention, and despite the gradual decline that occurred during the last three decades, it remained a serious health problem affecting adults, young children, and even infants [4]. Bottle-feeding caries, also known as ECC, was a serious public health issue. Research from all across the world has indicated that 46.2% of children have primary dental caries [5].

Dental caries is a biofilm-mediated, sugar-driven, multifactorial, dynamic disease that results in the phasic demineralization and remineralization of dental hard tissues. Caries can occur throughout life, both in primary and permanent dentitions, and can damage the tooth crown and, in later life, exposed root surfaces [6].

Given the importance of the host’s immune role in resisting Streptococcus mutans or other bacteria, the multiple differences in the patterns of human leucocytes antigen (HLA), which plays an important role in the body’s immune response, supports the role of genetic factors in disease susceptibility and infectious diseases resistance [7]. Many studies have shown evidence of the contribution of genetic factors in different ways to caries lesion occurrence, as the role that genes play is estimated between 40-60% [7,8].

HLA refers to the human leukocyte antigen system and it is a group of genes on chromosome 6p21.3 6 (a gene-dense region spanning approximately 4 Mb) [9]. Class I, II, and III polymorphic molecules are encoded by the chromosomal area known as the classical major histocompatibility complex (MHC), which includes HLA sites [8,10,11].

Class II polymorphic molecules include three loci DP, DQ, and DR, and all genes in the Class II region specifically have functions related to processing or presenting antigen to the T-cell receptor, which leads to a signaling process that leads to the proliferation of T cells helper, which leads to the stimulation of B cells, which produce antibodies to the antigen that triggered the response [8].

The best methods currently available for predicting future caries occurrence depend on whether the patient has had dental caries in the past or not. Therefore, it was necessary to investigate the possibility of finding an association between genetic factors and dental caries occurrence, which may provide us in the future with modern methods that enable us to investigate the risk of an individual for dental caries before the development of these lesions, which may save a lot of time and effort. Therefore, this study was conducted to predict ECC occurrence according to some HLA class II alleles (HLA-DR4, HLA-DQ2, HLA-DQ4).

## Materials and methods

Ethical considerations

The Ethics Committee of Damascus University, Syria's Scientific Research and Postgraduate Board accepted the study protocol (IRB No. UDDS-3290-27082018/SRC-1450). In addition to being requested to sign an informed consent form, the parent/guardian received a comprehensive information sheet prepared in simple, non-technical language beforehand. All methods were performed in accordance with the relevant guidelines and regulations.

Sample size determination

The Sample Size Calculation Program (PS Power and Sample Size Calculation Program, version 3.0.43) was used to calculate the sample size. 40 children per group was the minimum sample size needed to detect significant differences (significance level 5%, power 90%, effect size = 0.56), according to the sample size calculation.

Study population and inclusion criteria

80 children in all were assessed for the study and extended an invitation to take part based on the inclusion criteria that included being healthy children between the ages of three and six, not having a medical history of congenital or hereditary diseases, and not eating, drinking, or brushing their teeth for one to three hours prior to the tests. The children were divided into two groups: Group 1 (n = 40) (S-ECC children, deft ≥ 10), and Group 2 (n = 40) (free caries children, deft = 0).

DNA extraction

The study sample was collected from children attending the Faculty of Dentistry, Damascus University, Syria. The DNA tests were performed at Leishmania Research Center, Faculty of Pharmacy, Damascus University, Syria, and in the Department of Biology, Faculty of Science, Damascus University, Syria.

Initially, the children were instructed to perform a light mouth rinse, then they were asked to rinse a second time for 30 seconds or longer if possible and to spit into a sterile container before the buccal swab samples were taken. DNA samples were collected by taking a buccal swab using a sterile metal strip with blunt, rounded edges.

Buccal cell samples were transferred to the laboratory at the Leishmania Research Center, where DNA samples were extracted from them, using G-spin™ Total DNA Extraction kit (INtRON Biotechnology, USE) according to the following protocol:

All samples were shaken to ensure the presence of cells in the liquid before transferring them to the centrifugation tube to obtain the cells from the suspension, the samples were placed in the centrifugation (Hettich®, Germany) for one minute at 13,000 rpm/min, then the supernatant was disposed (the extraction procedures were carried out in 1 ml Eppendorf tube and the sample is large and placed in containers of 30 ml so that each 1 ml was centrifuged separately).

After obtaining the cell pellet, each of the following solutions was added in order: 200 μL of Buffer cl, 20 μL of Proteinase K, and 5 μL of RNase A. The mixture was then incubated in a hot water bath (JSR®, South Korea) at 56°C for 10-30 minutes, with the mixture being stirred every two minutes during the incubation period. Then 200 µL of Buffer BL solution was added to the mixture and incubated at 70°C for five minutes.

Then 200 microliters of absolute alcohol were added to the mixture and mixed. Then the contents of the Eppendorf tube were transferred to the tube provided with a filter to collect DNA. The new tube was centrifuged at a speed of 13,000/min rpm for one minute and the resulting contents were discarded after centrifugation to keep the DNA stuck in the filter.

Then 700 μl of Buffer WA solution was added and the tube was centrifuged for one minute at 13,000 rpm/min, then the filtered liquids were discarded and the same tube was reused by adding 700 μl of washing buffer (WB) solution and the tube was centrifuged for one minute at 13,000 rpm/min, then the filtered liquids were discarded and the same tube was reused with a second centrifugation procedure for the same tube for one minute to make sure the membrane was dry.

The filter was transferred to a new, sterile Eppendorf tube, and 30-100 μl of Buffer capillary electrophoresis (CE) solution was added directly on top of the membrane. The tube was incubated at room temperature for 1 min, then centrifuged at 13000 rpm/min for 1 min, after which the filter was discarded after the required DNA was deposited in the new tube. The concentration of the extracted DNA was measured before starting the polymerase chain reaction (PCR) using the NanoDrop™ device, where the obtained DNA yield was sufficient and appropriate to carry out the necessary reactions.

Polymerase chain reaction

PCRs were conducted to amplify specific regions of the DNA and detect the presence of the target genes HLA-DQ2, HLA-DQ4, and HLA-DR4 through specific primers (KAPABiosystems, ROCH, USA) as shown in Table 1, according to the following: 1) 0.5 μmol of each of the specific primers (HLA-DQ2- F, HLA-DQ2- R, HLA-DQ4-F, HLA-DQ- R, HLA-DR4-F, HLA-DR4-R, GH-F, GH-R),2) 150 nanograms of isolated DNA, 3) 12.5 µL of Master Mix (2X) Gene Direx® containing Taq DNA Polymerase, deoxyribonucleotides triphosphate (dNTPs), gel loading dye, fluorescent dye, and enzyme buffer.

**Table 1:** The results of reading the concentration of one of the DNA samples

Alleles	Sequencies
HLA-DQ2- F	GTGCGTCTTGTGAGCAGAAG
HLA-DQ2- R	TGCAAGGTCGTGCGGAGCT
HLA-DQ4-F	GTGCTACTTCACCAACGGGACC
HLA-DQ- R	CTGGTAGTTGTGTCTGCATACG
HLA-DR4-F	CGTTTCTTGGAGCAGGTTAAACA
HLA-DR4-R	CTGCCGCTGCACTGTG
GH-F	GCCTTCCCAACCATTCCCTTA
GH-R	TCACGGATTTCTGTTGTGTTTC

Finally, a sufficient amount of nuclease-free water was added to the reaction to reach a final volume of 25 μl, within three separate reactions for each sample, and in the presence of amplified primers for a reference gene that is naturally present within DNA, which is the GH. The thermocycler is set at the appropriate reaction conditions (Table 2).

**Table 2:** Thermocycler settings

Settings		Temperature	Time
Start		94° C	5 minutes
Cycles		-	35 cycles
Denaturation		94° C	30 seconds
Annealing	HLA-DQ2	56° C	45 seconds
	HLA-DQ4	58° C	
	HLA-DR4	60° C	
Extend		72° C	1 minute
Final extensions		72° C	5 minutes

The denaturation temperature of the initial DNA strips was set at 94°C for five minutes, followed by 35 cycles, each consisting of denaturation at 94°C for 30 seconds, followed by a suitable temperature for the binding of primers for 45 seconds during which the primers bind to their complementary sequences (The ideal temperature was chosen after conducting several reactions of the polymerase chain with gradient PCR temperatures until adjusting the appropriate settings for each gene). This was followed by extending at 72 °C for one minute, and after the end of the fortieth thermocycling, the device was set at 72 °C for 5 minutes to complete the final extensions of the DNA strips.

Analysis of the results of PCR reactions using electrophoresis

The 1.5% agarose gel was prepared by adding 1.5 g of Agarose-Molecular Biology Grade (Genedirex®, Taiwan) with 1 ml of TAE 50 X (BDH Laboratory®, England) and completing the volume to 100 ml of distilled water. The mixture was heated in the microwave (Triview®, China) with stirring until the agarose was completely dissolved and a clear solution was obtained. Then the solution was poured into a special mold to prepare the gel after it cooled slightly, as 5 microliters of ethidium bromide (final concentration 0.5 μg/ml) (Biobasic®, UK) was added, then the comb was placed to form the sample injection wells and the mixture was left until the gel completely polymerized (gelatinize).

 The gel was transferred to the electrophoresis basin and flooded with 1X TAE solution as an electrophoresis buffer, then the comb was removed and the products of the PCR reactions were injected sequentially into the gel wells starting with DNA Ladder (Genedirex®, Taiwan) consisting of 12 fragments, with the following lengths: 100, 200, 300, 400, 500, 600, 700, 800, 900, 1,000, 1,500, 3,000 strands of bases.

**Figure 1:**
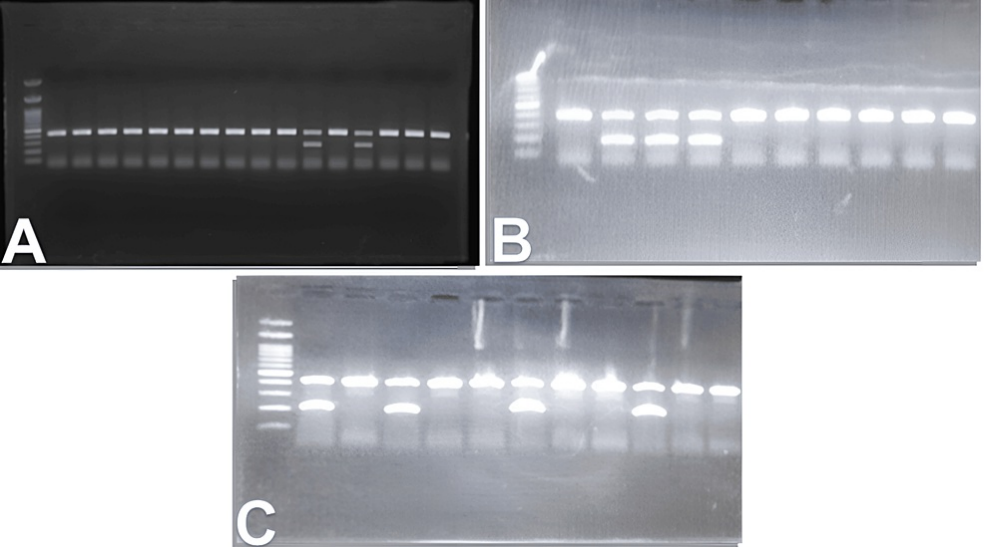
Electrophoresis results of some samples (A) DR4 gene, (B) DQ2 gene, (C) DQ4 gene

The wells were equipped with 5-10 μl of DNA subject to the PCR reaction, in addition to a basic well containing 5 μl of the ladder solution. The electrophoresis was set to 100 mA, with a potential difference of 115 V, for 20 minutes. DNA strands were shown after electrophoresis, by exposing the gel to ultraviolet light (UV), and the gels were photographed to document the results using a gel documenter (Figure 1).

Statistical analysis

The SPSS 21.0 program (IBM, Armonk, USA) was used to conduct the statistical analysis. The chi-square test was used to examine the data. The experiment was performed at α=0.05.

## Results

As shown in Table 3, the study sample comprised 80 children of both sexes (56.3% males, 43.7% females), ages ranging from three to six years old (4.5 ± 1.0). They were all attending the Faculty of Dentistry at Damascus University during the period from 2021 to 2022, and they were divided into two main groups according to the incidence of dental caries (severe early childhood caries (S-ECC) children, and caries-free children).

**Table 3:** Basic sample characters

Gender		Ages			
Male	Female	Min	Max	Mean	SD
45 (56.3%)	35 (43.7%)	3	6	4.5	1.0

**Figure 2:**
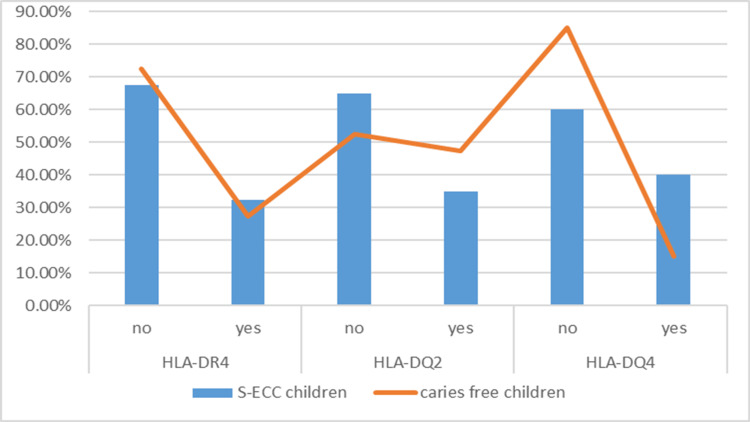
The percentage of presence of some of the studied alleles according to the incidence of S-ECC S-ECC: Severe early childhood caries

The chi-square test was applied and the odds ratio was calculated to study the independence of each of the studied alleles from the S-ECC variant (Table 4 and Figure 2).

**Table 4:** The independence of the studied alleles from the incidence of dental caries (S-ECC) S-ECC: Severe early childhood caries

Studied allele	Presence of the studied allele	N		Chi-value	P-value	Odds ratio	The confidence level of odds ratio	
		S-ECC children	Caries-free children					
HLA-DR4	Yes	27 (67.5%)	29 (72.5%)	0.238	0.626	1.269	0.487	3.311
	No	13 (32.5%)	11 (27.5%)					
HLA-DQ2	Yes	26 (65.0%)	21 (52.5%)	1.289	0.256	0.595	0.242	1.462
	No	14 (35.0%)	19 (47.5%)					
HLA-DQ4	Yes	24 (60.0%)	34 (85.0%)	6.270	0.012	3.778	1.291	11.057
	No	16 (40.0%)	6 (15.0%)					

Table 4 shows that there were no statistically significant differences in the frequency of occurrence of S-ECC, HLA-DR4, and HLA-DQ2 alleles (p = 0.626, 0.256 respectively), while the incidence of S-ECC was associated with the DQ4 allele (p = 0.012). The odds ratio was 3.778, and it is noted from Figure 2 that the S-ECC children who did not have the HLA-DQ4 allele were smaller free caries children who had the DQ4 allele, and therefore the presence of the HLA-DQ4 allele in the child increases the chance of developing dental caries.

## Discussion

When looking at the best methods currently available to predict the future incidence of caries, it depends on whether the patient has had tooth decay in the past or not. As a result, it was imperative to concentrate on the preventive the potential to predict dental caries before they occur, which reduces the need for therapeutic interventions, particularly in age groups that are difficult to deal with, such as children, and if left untreated, it will have negative effects on public health and the child's quality of life [12]. This increase in the need for indicators to assess the risk of caries is a result of the increasing focus on early interventions as the cornerstone of strategies directed towards the prevention of dental caries. In light of the foregoing, this study was carried out to try to reach a link between genetic factors and dental caries infection.

It is known that multiple factors contribute to a person's risk of developing and developing caries. Besides environmental factors (diet, oral care, etc.), and factors related to the oral condition of the host person, genetic factors have been shown to play an important role in the etiology of dental caries [13]. Several previous studies have identified an association between several genes and dental caries [14], including genes related to enamel formation such as AMBN, AMELX, and ENAM [15]; taste receptor genes such as TAS2R38, TAS1R2 [16,17]; immune-related genes such as HLA [18]; and saliva genes such as MUC5B, PRH1 [19,20].

Dental caries is affected by a complex interaction of genetic and environmental factors. Multiple studies have focused on the gene patterns of the HLA, which regulates the immune response to foreign antigens [21]. The choice fell on the HLA genes, which are responsible for determining the immune host response, because of the role of these genes in disease susceptibility and resistance to diseases [8]. Streptococcus mutans is present in almost all individuals, but the significant differences in levels of oral colonization between individuals may be explained by changes in immune responses. Some types of histocompatibility complexes stimulate B lymphocytes less effectively than others and will therefore produce lower levels of IgA against streptococcus mutans [22].

The study sample consisted of 80 healthy children with similar ages ranging from 24-71 months since ECC affects the primary teeth of young children under six years of age [23]. The caries severity was recorded according to the deft index for primary teeth, as it is the most widely used in the world it has been used for more than 66 years due to its multiple uses in assessing dental caries and giving it a realistic picture of the health culture related to diets and oral health care procedures [24]. It is also distinguished by its ease of use, and simplicity, and is the first standard in the world that provides a unified research methodology regarding the prevalence and severity of dental caries in different age groups [25]. The sample was divided into two equal groups, as the control group was chosen to be healthy from dental caries with deft=0. While experminteal group was chosen with deft>10 so that there was a clear discrepancy between the two samples leading to more clear and distinct results.

DNA samples were collected by taking a buccal swab, which is faster than blood samples, and less aggressive, in addition to the possibility of applying them to young ages [26]. The buccal swab was done using a sterile metal strip with blunt edges to increase the number of cells collected, where the cells obtained in this way were greater than the cells using the sterile cotton swab method.

This study showed a positive association between the DQ4 allele and an increased chance of developing S-ECC, while no statistically significant correlations appeared between the DR4 and DQ2 alleles and developing S-ECC. The results of the current study agreed with Ruihan's study, which concluded that there is an association between the HLA gene and the susceptibility of children to developing dental caries, as it found that the DQB1 * 02 allele may represent a protective factor from dental caries, while DQB1 * 05 allele may be responsible for developing dental caries [27]. Moreover, Ozawa et al.'s study also showed that HLA-DQB1*0601 was associated with the lactobacilli numbers of mutans streptococci and suggested that HLA class II alleles may be related to the salivary populations of oral microorganisms.

The results of this study differed from Valarini et al.'s study which found that Brazilian adolescents positive for HLA-DQ2 allele were less likely to have dental caries, and there was no statistical difference between HLA-DR4, -DQ4, -DQ5, -DQ6 and dental caries. This difference can be attributed to the difference in age group, as this study was conducted among children, in addition to the difference in race studied [28]. Moreover, the results of this study differed from Wang et al study found that HLA-DRB1*13 allele frequency was significantly higher in patients with caries while HLA-DRB1*09 allele was significantly lower in patients with caries than in healthy controls [29]. This difference can be attributed to the difference in the studied community. Bagherian et al.'s study also demonstrated a significant increase in the frequency of HLA-DRB1 in patients with ECC, while the frequency of HLA-DQB1 alleles was not significantly different between patients with or without ECC [18].

One of the main limitations of this study is that it did not examine the other HLA subtypes or the geographic region, which could explain the results of this study more easily, because the caries patients were from a different geographical region, even within the city, associated with a different ethnical group. While the sample size in this study was good, we suggest that larger sample sizes and other HLA subtypes (such as HLA-DQB1) be studied in future research to validate these findings.

## Conclusions

Within the limitations of this study, it may be concluded that HLA class II molecules may play a crucial role in predisposing to ECC among Syrian children. A potential association between HLA alleles and caries susceptibility were found, since positive HLA-DQ4 may enhance the chance of developing ECC. However, no association was found between HLA-DR4 and HLA-DQ2, and ECC.
